# Supplementary material for: Egg clutch dehydration induces early hatching in red-eyed treefrogs, Agalychnis callidryas
Source: PeerJ. 2017 Jul 14;5:e3549. doi: 10.7717/peerj.3549 (PMC5511700; doi:10.7717/peerj.3549)
Supplement: File S1 [file peerj-05-3549-s002.rtf]

Survival & hatching by clutch dataClutch = number assigned to each clutch  Clutch size = number of eggs of each clutchTreatment = the hydration treatment (spraying regime) assigned to each clutch Eggs that survived to hatch  = number of eggs of each clutch that survived to hatch % hatching success = mean percent of embryos that hatched successfully Start hatching (hours) = time in hours for the first time point when any eggs had hatched ≥Half hatched (hours) = time in hours when 50% or more of the eggs (that eventually hatched) had hatchedFinished hatching (hours) = time in hours when all the eggs (that would eventually hatch) had hatched Cumulative hatching dataClutch = number assigned to each clutch  Treatment = the hydration treatment (spraying regime) assigned to each clutch Hours from oviposition = time in hours, measured from midnight of oviposition, when clutches were checked for hatching during the experiment Days from oviposition = age in days, measured from midnight of oviposition, when clutches were checked for hatching during the experimentCumulative proportion of hatched = cumulative proportion of eggs hatched of all that eventually hatched Eggs hatched = total number of eggs hatched Egg diameter and clutch thickness dataClutch = number assigned to each clutch  Treatment = the hydration treatment (spraying regime) assigned to clutches  Age(days) = embryos’ age, determined by number of days after oviposition Diameter (mm) = mean egg size per clutch, calculated from two orthogonal diameters measured, in millimeters, for each of 10 eggsThickness (mm) = measurement of thickness of each clutch (millimeters) 
